# Synthesis, characterization and electrochemical properties of some biologically important indole-based-sulfonamide derivatives

**DOI:** 10.1186/s13065-020-00691-5

**Published:** 2020-05-27

**Authors:** Mohamed Ibrahim, Muhammed Taha, Noor B. Almandil, Abdel-Nasser Kawde, Muhammad Nawaz

**Affiliations:** 1 Department of Clinical Pharmacy Research, Institute for Research and Medical Consultations, Imam Abdulrahman Bin Faisal University, P.O. Box 1982, Dammam, 31441 Saudi Arabia; 2 Chemistry Department, College of Sciences, King Fahd University of Petroleum and Minerals, Dhahran, 31261 Saudi Arabia; 3 Department of Nano-Medicine Research, Institute for Research and Medical Consultations, Imam Abdulrahman Bin Faisal University, P.O. Box 1982, Dammam, 31441 Saudi Arabia

**Keywords:** Synthesis, Indole sulfonamide derivatives, Voltammetry, Oxidation, Electrochemical parameters

## Abstract

A new series of indole-based-sulfonamide derivatives (**A1**–**A8**) was synthesized by treating 5-fluoro-1H-indole-3-carbohydrazide with different aryl-sulfonyl chloride in the presence of pyridine. All synthesized derivatives (**A1**–**A8**) were characterized by different analytical methods. The electrochemical behavior of these compounds (**A1**–**A8**) was investigated in detail using cyclic voltammetry (CV) and square wave voltammetry (SWV) at the pencil graphite electrode (PGE). In the present study, the redox behavior of all derivatives varies due to the nature of substitutions in the indole sulfonamide moiety. Various fundamental electrochemical parameters, including the standard heterogeneous rate constants (k_s_), and the electroactive surface coverage (*Г*) were calculated from the obtained CVs. The obtained results shed light on the understanding of structure–activity relationships of this class of compounds.
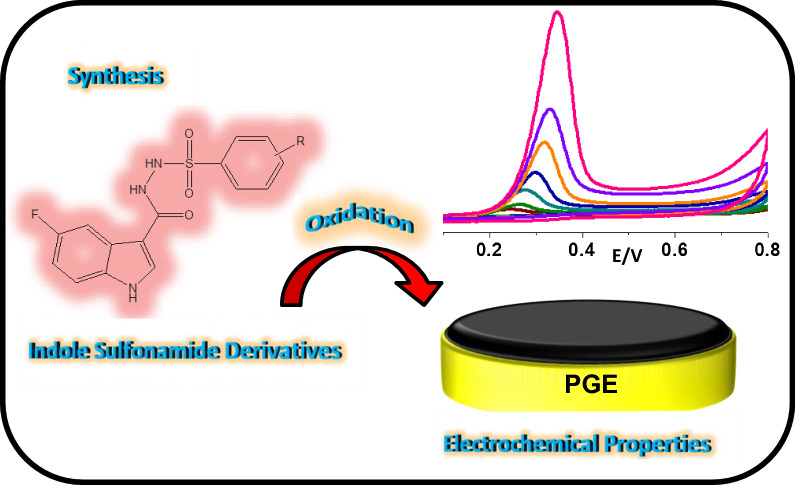

## Introduction

Indole ring exists in several naturally occurring alkaloids [1]. Indole derivatives were reported to have biological effects [2], anti-inflammatory [3], antitubercular [4], and antimicrobial [5] activities. Thio-indole based analogs act as antifungal [6], antimicrobial [7], antibacterial, analgesic [8, 9], anticonvulsant [10], antioxidant [11], antidepressant [12], antihypertensive [13], and antiviral agents [14]. Indole sulfonamide undergoes substitution, mainly at the C3 position, possess hydrophilic features like sulfonyl group, and is regarded as an appropriate pharmacophore equivalent for replacing active sites in drug design [15–17]. Consequently, the redox properties of the indole sulfonamide may be very useful for their efficient uses. It is well known that the electroactive indole derivatives are easily oxidized at carbon-based electrodes [18]. Accordingly, the knowledge of the electrochemical oxidation of these compounds is helpful to understand their action mechanism as well as pharmacokinetic and pharmacodynamic purposes in chemical and biological processes, if they find use as drugs [19]. The electrochemical techniques have been effectively applied to study the redox properties of several biologically important compounds [20, 21]. As reported previously, the redox behavior of indole derivatives was investigated using various electrodes: pyrolytic carbon [22], glassy carbon [17, 23], platinum [24], lead dioxide [25] boron-doped diamond [26, 27], and other electrodes [28–30]. Among the studied indole compounds with a substituent at C3, the results of electrochemical oxidation of indole-3-acetic acid, indole-3-propionic acid, indole-3-butyric acid, indol-3-acetamide, tryptamine, gramine and tryptophan were reported for the measurements done with the usage of a glassy carbon electrode as the working electrode [23, 31]. The oxidation behavior of indole derivatives in aqueous solutions is more complex and suggested an irreversible pH-dependent process. Two oxidation peaks were found, the first peak corresponds to the oxidation at position C2 on the pyrrole ring and the second peak to electrochemical hydroxylation at the position C7 on the benzene moiety. Herein, we report the synthesis of eight biologically important indole-based-sulfonamide compounds with a different aryl-sulfonyl substituent at C3. Furthermore, the electrochemical properties for these new compounds were investigated using CV and SWV techniques at PGE. Based on the obtained results, the redox behavior was proposed to highlight important aspects of structure–activity relationships of this class of compounds.

## Result and discussion

### Synthesis of indole-based-sulfonamide derivatives

**Scheme 1:**
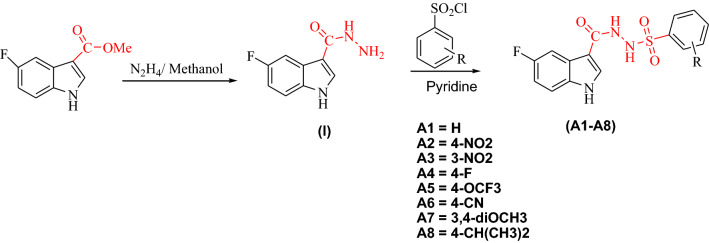
Synthesis of indole-based-sulfonamide derivatives **A1**–**A8**

The synthesis of indole-based-sulfonamide derivatives (**A1**–**A8**) began with the synthesis of 5-fluoro-1H-indole-3-carbohydrazide (I) which was synthesized by heating under reflux methyl 5-fluoro-1H-indole-3-carboxylate with mixture of hydrazine and methanol. 5-Fluoro-1H-indole-3-carbohydrazide (I) reacted with different aryl sulfonyl chloride in the presence of pyridine. All synthesized compounds (**A1**–**A8**) were fully characterized by different spectroscopic methods Scheme 1.

### Electrochemical properties of Indole-Based-Sulfonamide Derivatives

#### Cyclic voltammetric studies

**Fig. 1:**
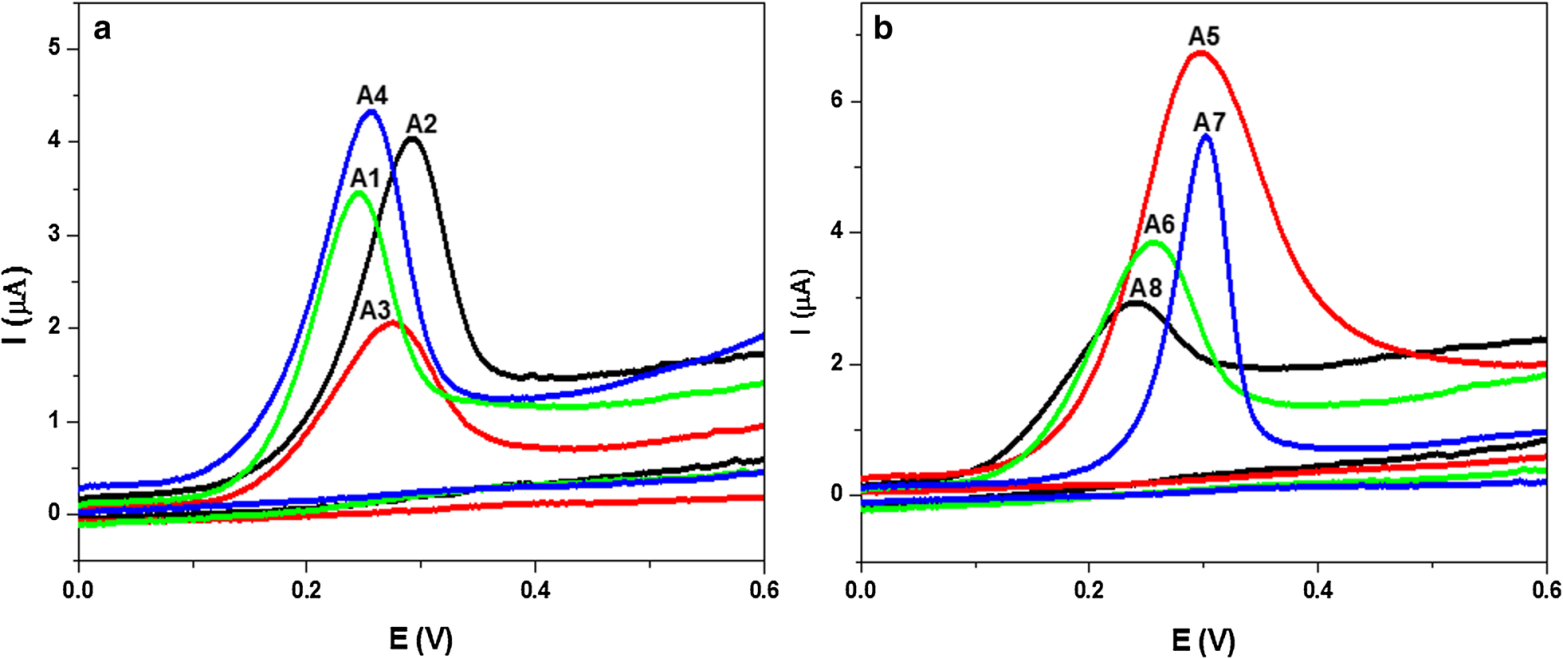
CVs of 3 × 10^−5^ M of indole sulfonamide compounds (**a**) **A1–A4** and (**b**) **A5–A8** in PBS of pH 7.4 obtained at PGE scan rate, 0.10 Vs^−1^

The electrochemical properties of the indole sulfonamide derivatives (**A1**–**A8**) were studied in comparison to the parent (**A1**) using CV. The CVs of 3 × 10^−5^ M of the investigated compounds (**A1**–**A8**) recorded in 10% aqueous ethanol (as optimum ethanol/H_2_O ratio) at pH 7.4 using pencil graphite electrode (PGE) as working electrode, Ag/AgCl as reference electrode and Pt wire as counter electrode at ambient temperature (Figs. 1a, b). The overlapping CV behavior of all compounds have come through the existence of the only one well-defined oxidation peak within the investigated potential range (from 0.0 to + 0.8 V). A well-known anodic peak (P_a_) was developed from the oxidation of the analyte species in the positive scan for all compounds however; no cathodic peak/s was observed in the negative potential scan revealed the stability of the analyte species to reduction. The presence of a sole oxidation peak for all compounds suggests an irreversible electrochemical process. However, the values of E_pa_ − E_pa/2_ between 50 and 70 mV (Table 1) confirmed the irreversibility of the electrochemical oxidation process.

**Table 1:** Summary of results obtained from CV studies (pH 7.4)

Comp.	R	E_pa_ (mV)	E_pa_ − E_pa/2_ (mV)	*d* log I_pa_/*d* log *v* (µA/mV s^−1^)	αn	K_s_ × 10^3^ (s^−1^)	Г × 10^−11^ (mol cm^−2^)	Г × 10^13^ (molecule cm^−2^)
**A1**	H	240	47	0.67	1.00	6.04	6.94	4.17
**A2**	*4*-NO_2_	282	57.1	0.75	0.864	17.39	8.09	4.87
**A3**	*3*-NO_2_	274	65.1	0.72	0.851	6.88	5.62	3.38
**A4**	*4*-F	258	56.1	0.66	0.976	3.98	9.75	5.87
**A5**	*4*-OCF_3_	296	65.1	0.66	0.937	6.02	25.80	15.54
**A6**	*4*-CN	264	63.1	0.78	0.826	5.24	8.18	4.93
**A7**	*3,4*-diOCH_3_	302	32.1	0.78	1.210	9.98	9.01	5.43
**A8**	*4*-CH(CH_3_)_2_	234	73.1	0.75	1.200	4.72	4.63	2.79

**Fig. 2:**
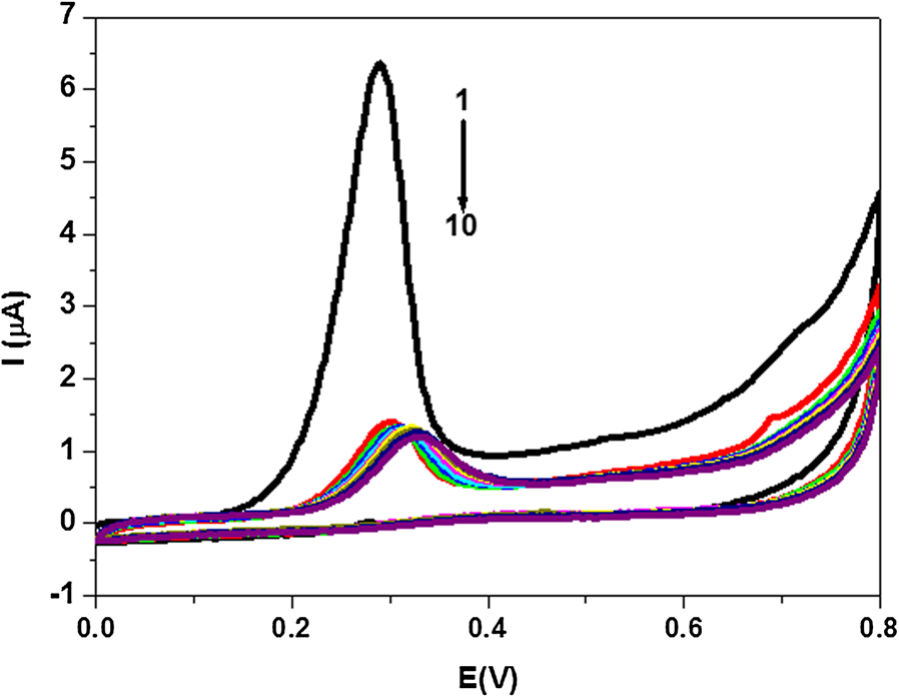
CVs (scan 1–10) of 3.0 × 10^-5^ M of **A4** in PBS of pH 7.4 obtained at PGE scan rate, 0.10 Vs^−1^

**Fig. 3:**
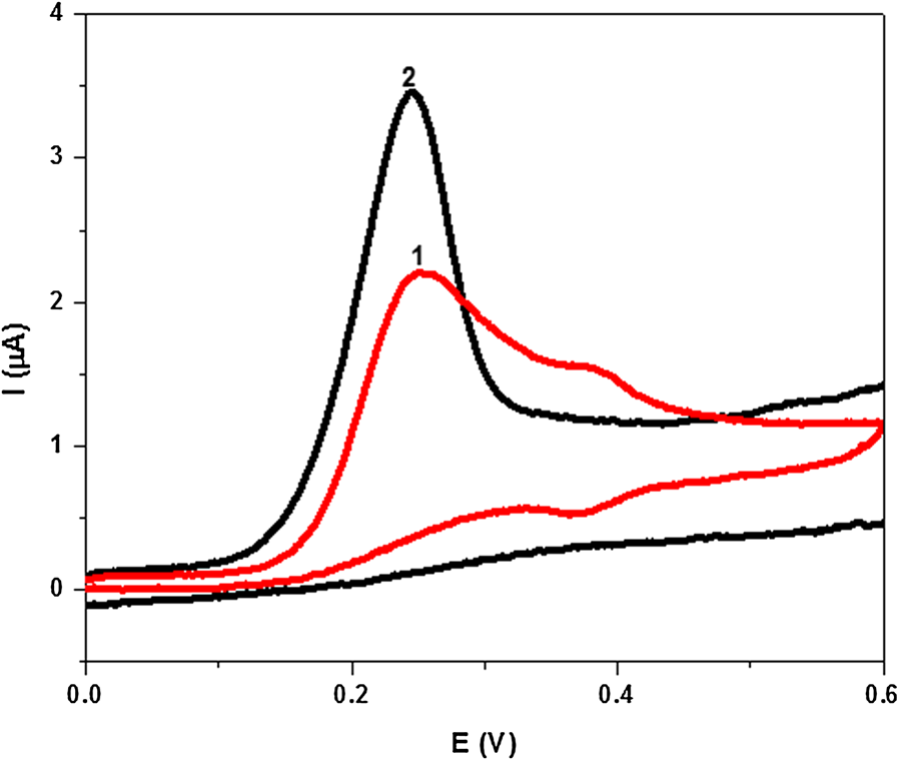
CVs of 3x10-5 M of 5-fluoro-1H-indole-3-carbohydrazide (**1**) and compound **A1** (2) in PBS of pH 7.4 obtained at PGE scan rate, 0.10 Vs^−1^

The electrochemical oxidation active center of indole derivatives is the indole moiety, however the reduction of the sulfonyl group (–SO_2_–) is very difficult to attain [23, 32]. Figure 2 recorded the successive CV scans of **A4** in the same analyte solution without changing the PGE surface. A significant decrease in peak height was observed with subsequent scans indicating that the oxidized products are strong adsorbed on the surface of PGE, hence blocking it for further sensing of the analyte. Similar electrochemical behavior was also observed for the other compounds (Additional file 1: Fig. S1). To support the working hypothesis the indole moiety in **A1**–**A8** that undergoes oxidation, the oxidation behavior of **A1** was compared with a model compound. As model substances for the oxidation of indole ring, the 5-fluoro-1H-indole-3-carbohydrazide (**I**) was used. The potential range, in which the model compound (**I**) is oxidized is comparable to that observed for oxidation in sulfonamide compound (**A1**) (Fig. 3). A small couple appears at 275 mV for (**I)**, may be due to adsorption complications. By comparing the chemical structure of **(I)** with that of **A1**, it is observed that **A1** contains additional substitution by benzosulfone moiety. Such substitution should not influence the mechanism of the electrochemical oxidation process. These results indicated that the potential at which the oxidation occurs is strongly dependent on the nature, position and number of substituents on benzene ring of the investigated compounds (Table 1). As reported previously [33–35], the oxidation of indole compounds, with a substituent at C3 position, in aqueous solutions is more complex and suggested an irreversible pH-dependent process. Also, it is well known the benzene ring of indoles is less reactive than the pyrrole ring, since the oxidation reaction for all indole derivatives (**A1**–**A8**), which present mainly one oxidation peak at the potential range from 240 mV to 300 mV, corresponds to the oxidation at C2 position on the pyrrole ring [23, 31, 36].

#### Effect of pH

CVs of the compounds under investigation were also recorded in phosphate buffer solution (PBS) at different pH values (from pH 3 to pH 11). The results indicated that, in acidic buffer solution the oxidation peak potential appeared at higher positive values and shifted to lower values with an increase in pH, which indicating that the electron transfer process is more easily in neutral and basic buffer solution. It is apparent that below pH 7 the -NH group of indole moiety is protonated to a great extent and therefore presumably the oxidation of compounds A is more difficult. These results suggest the involvement of H^+^ ion in the electron transfer process (Additional file 1: Fig. S2) [37]. Many studies reported that organic compounds showing pH-dependent oxidation undergo deprotonation reaction through oxidation [37, 38]. As shown in Table 2, the shifts of peak potential (E_pa_) with pH for all indole sulfonamide derivatives are linear with slopes in the range from 54 mV to 66 mV (R from 0.993 to 0.999). These data (the slope of ∼ 59 mV per pH unit) suggested that the electrochemical oxidation processes of the investigated compounds include the same number of protons and electrons [38]. Depend on the equation dE_p_/dpH = 0.059*x*/αn, the number of proton (*x*) was calculated to be ~ 1. In this manner, the oxidation reaction of the investigated compounds on the surface of PGE is a one-proton and one-electron irreversible process.

**Table 2:** Data of the linear plot of E_pa_ vs. pH (E_pa_ (V) = *a* + *b* pH) for all indole sulfonamide compounds at different pH values using CV

Compound	R	*a* (intercept)	*b* (slope)	Regression coefficient
**A1**	H	0.732	0.063	0.998
**A2**	*4*-NO_2_	0.803	0.069	0.998
**A3**	*3*-NO_2_	0.767	0.066	0.994
**A4**	*4*-F	0.766	0.065	0.999
**A5**	*4*-OCF_3_	0.792	0.067	0.995
**A6**	*4*-CN	0.770	0.066	0.993
**A7**	*3,4*-*diOCH*_*3*_	0.671	0.054	0.996
**A8**	*4*-CH(CH_3_)_2_	0.693	0.059	0.995

#### Effect of substitutions

The redox data for the compounds **A2**–**A8** were compared to those of parent **A1** and summarized in Table 1. It was found that, the oxidation peak potential of these compounds **A2**–**A8** varies, because of the different substituent in the indole moiety. The appearance of the oxidation potentials of compounds **A2**–**A8** at more positive potential values than the parent **A1** (240 mV), indicated the most difficult electron transfer process due to the electron-withdrawing nature of substituents. It can be observed that oxidation potentials of the compounds **A2**–**A8** increase with the increasing degree of electron-withdrawing groups, as noticed between **A3** (*3*-NO_2_) and **A4** (*4*-F), or between **A4** (*4*-F), and **A5** (*4*-OCF_3_). Compared to the parent compound **A1**, compound **A8** (*4*-CH(CH_3_)_2_) has the lowest Ep_a_ (234 mV) among the all derivatives listed, and compound **A7** (*3,4*-*di*OCH_3_) has the highest Ep_a_ (302 mV) due to its two electron-withdrawing substituents. Easily oxidation of **A8** is attributed to the electron-donating effect of the isopropyl group (*4*-CH(CH_3_)_2_). This is as expected because electron-withdrawing groups increase Ep_a_ of a compound by removing electron density from the π system and thus more energy is needed to remove an electron, whereas electron-donating substituents decrease the oxidation potential. The electrochemical behavior of these compounds indicated that by varying the electronic properties of the substitution the anodic peak potential of the electrophore can be modified.

#### Effect of scan rate

**Fig. 4:**
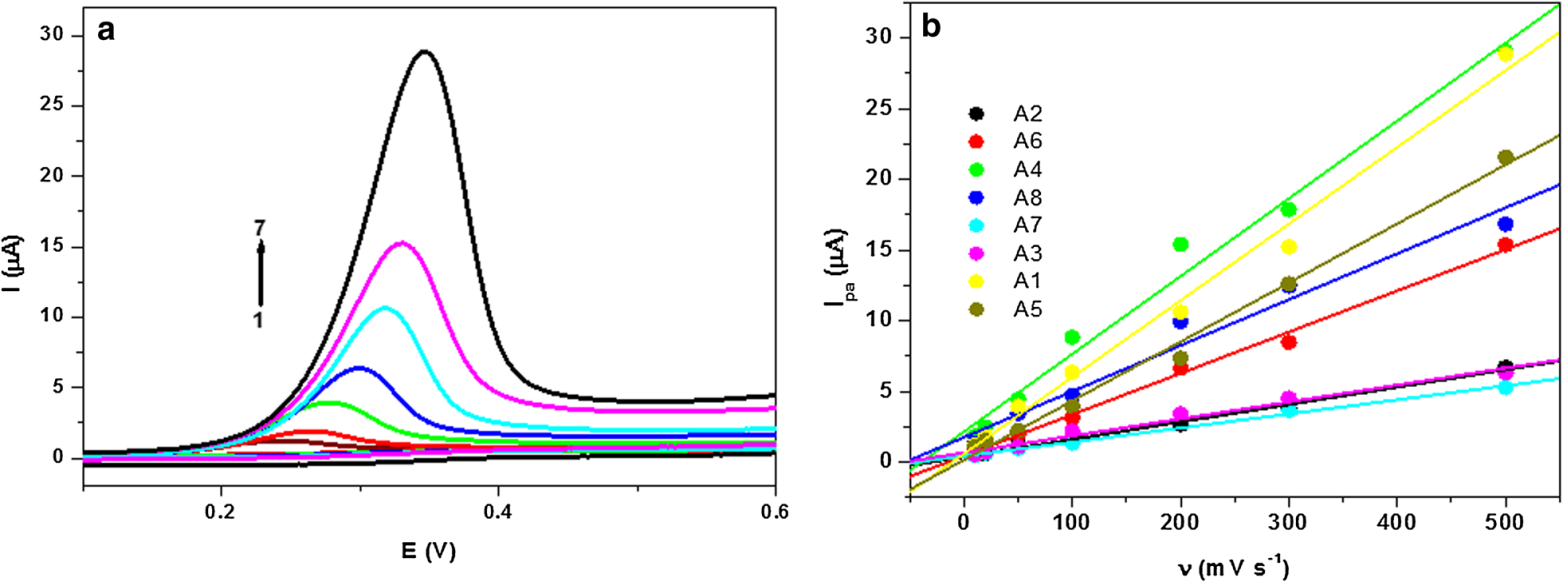
**a** CVs of 6.5 × 10^−5^ M of **A1** on surface of PGE at various scan rates; (1) 10, (2) 20, (3) 50, (4) 100, (5) 200, (6) 300 and (7) 500 m Vs^−1^ in PBS of pH 7.4. **b** Plot of I_pa_ (µA) vs. ν (m V s^−1^)

**Scheme 2:**
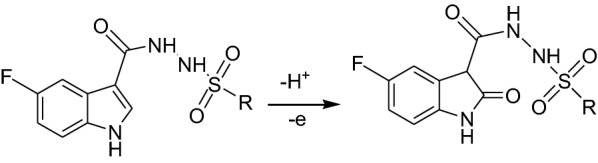
Proposed oxidation mechanism of indole sulfonamide derivatives at PGE

The dependence of the anodic electrochemical process for all indole sulfonamide derivatives on the scan rate was examined at pH 7.4 at various sweep rates (Fig. 4). As shown in Fig. 4a by increasing the scan rate (*v*), the anodic peak potential (E_pa_) shifted toward the positive direction which confirms the irreversibility of the electrochemical process of indole sulfonamide derivatives [39]. Experimental plots of I_Pa_ vs. ν are shown in Figs. 4b. It was found that the peak currents increased linearly with the scan rate, proposing the electrochemical reactions were an adsorption-controlled step rather than a diffusion-controlled process [40]. This result was also confirmed from the data analysis through a plot of log I_pa_ vs. log ν, which reveals slope values in the range from 0.62 to 0.78 (Additional file 1: Fig. S3 and Table 1). These data propose a mainly adsorption-controlled mass transfer process [41]. As for an irreversible electrode process, the Laviron equation [42], was used to calculate αn and k_s_ values as follows:

$${\text{E}}_{\text{pa}} = {\text{ E}}_{\text{o}} + \, \left( {{\text{RT}}/\upalpha{\text{nF}}} \right){ \ln }\left( {{\text{RTk}}_{\text{s}} /\upalpha{\text{nF}}} \right) \, + \, \left( {{\text{RT}}/\upalpha{\text{nF}}} \right){ \ln }\upnu$$

where k_s_ is the standard heterogeneous electron transfer rate constant of the surface reaction, α is the electron transfer coefficient, ν is the scan rate, E_o_ is the formal redox potential and n is the electron transfer numbers. Other symbols have their common meanings. The E_o_ value at PGE can be deduced from the intercept of E_pa_ vs. ν plot on the ordinate by extrapolating the line to ν = 0. Thus, the values of αn and k_s_ (s^−1^) for all investigated compounds were obtained from the slope and intercept of the linear plot of E_pa_ vs. ln ν, respectively and tabulated in Table 1 (Additional file 1: Fig. S4). Where, α was supposed as 0.5, for an irreversible electron transfer, the n was calculated to be ~ 1 which suggested that one electron was involved in the oxidation process. These results indicate the direct electron transfer on the surface of the PGE. The k_s_ values with an order of 10^3^ s^−1^ characterize the indole sulfonamide oxidation process to be irreversible. Furthermore, the higher values of k_s_ showing faster electron transfer kinetics at pH 7.4 can be ascribed to the closest approach of indole sulfonamide at the surface of PGE due to the probable compression of the electrical double layer under these conditions. In this context, the electrochemical reaction process for the indole sulfonamide derivatives in aqueous solution (pH 7.4) at PGE occurs with the transfer of one electron and one proton and may be written as shown in scheme 2. This reaction mechanism in agreement with the previous studies [23, 31, 36]. These results displayed that the electrochemical oxidation of indole compounds with a substituent at C3 position occurs with a similar mechanism, with the only differences in the currents and the oxidation potentials due to the different substituent in C3 position of the pyrrole ring which does not change the oxidation pathway. Moreover, indole containing compounds are oxidizable derivatives, explanation of their oxidation pathways can introduce a model for the mechanism study of electron exchange between indole-containing enzymes or proteins and solid surfaces, and can be used for constructing new biomedical and biosensors devices.

The surface coverage for all sulfonamide compounds (Г, mol cm^−2^) was further estimated from the calculated charge (*Q*) under the reductive CV peak. Where the adsorbed amount of the investigated compounds is proportional to the total charge (*Q*) consumed during the oxidation of adsorbed molecules according to equation [43]:

$$Q = \mathop \int \limits_{{E_{s} }}^{{E_{e} }} i_{s} dE = nFA{{\varGamma }},$$

where *Q* is the charge involved in the electrooxidation process, n is the number of electrons, F is the Faraday constant and A is working electrode surface area. The Γ values of the investigated compounds at PGE were estimated and listed in the Table 1. The results showed that the 4-substiuted sulfonamide derivatives exhibit the largest tendency for specific adsorption.

The magnitude of the Г decreases in the order **A5** > **A4** > **A7** > **A6 **> **A2 **> **A1 **> **A3 **> **A8**. This can be easily expected by the effect of the specific adsorption of 4-substituted withdrawing groups (**A2**, **A4**–**A7**) compared to 3-substituted withdrawing group (**A3**) or 3-substituted donating groups (**A8**). On the other hand, the Г value for compound **A1** (R = H) was found to be less than 4-substituted compounds and greater than 3-substituted compounds. The large value of Γ for **A5** confirmed that the presence of *4*-OCF_3_ strongly affected the adsorption character of this sulfonamide compound compared to other substitutions, which proposing that the **A5** molecules assume a perpendicular orientation at the electrode surface due to the enhance of the strength of stacking interactions between adjacent molecules. The results also indicate that the degree of adsorption of sulfonamide derivatives is influenced not only by the nature of the substitution but also by the position of this substitution.

#### Square wave voltammetry

**Fig. 5:**
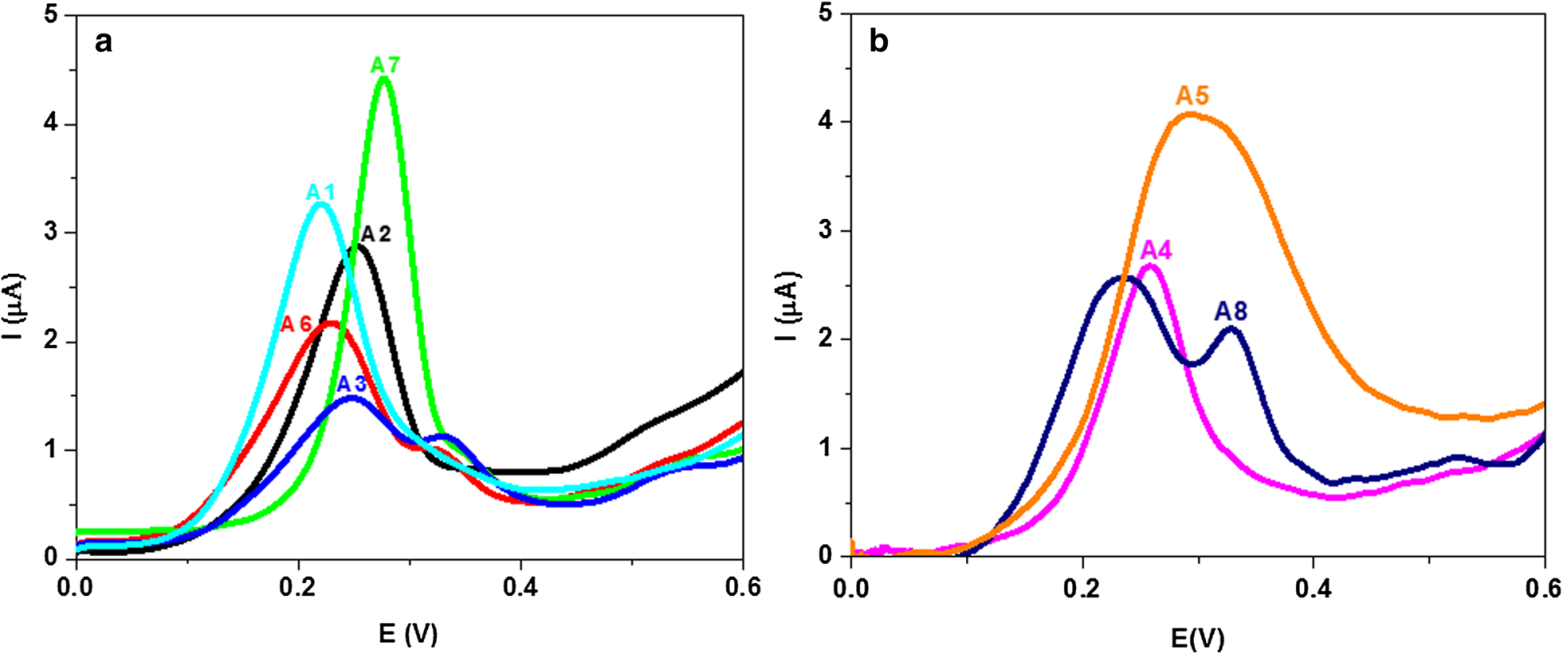
SWV of 3 × 10^−5^ M of **A1**, **A2**, **A3**, **A6** and **A7** (**a**) and **A4**, **A5** and **A8** (**b**) at PGE in PBS at pH 7.4. Accumulation potential, 0.0 V; accumulation time, 60 s; scan increment, 6 mV; frequency, 50 Hz and pulse height, 25 mVpp

**Fig. 6:**
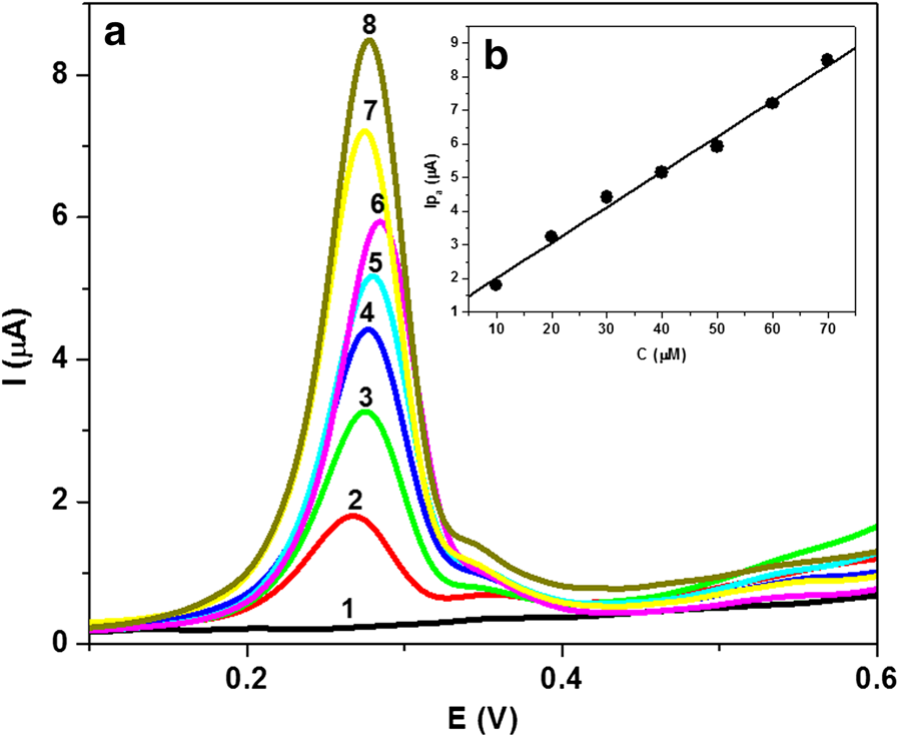
**a** SW voltammograms of **A7** at PGE in PBS at pH 7.4. [**A7**]: (1) blank, (2) 10, (3) 20, (4) 40, (5) 50, (6) 60, (7) 70 and (8) 90 μM. **b** Calibration plot of I_pa_ (μA) vs. [**A7**] in PBS of pH 7.4. Other conditions as in Fig. 5

The SWV behavior of the compounds under investigation was also studied in PBS (10% ethanol) of pH 7.4 at PGE (Fig. 5). Like a CV, an oxidation peak of **A1**–**A8** was registered which attributed to the oxidation of carbohydrazide group. It was found that, the SWV oxidation peak potential of these compounds **A1**–**A8** shifted to more positive potential in the sequence: **A8** > **A1** > **A4 **> **A6 **> **A3 **> **A2 **> **A5 **> **A7.** As expected, these results indicate that the electron-withdrawing groups shifted the SWV oxidation peak to a more positive potential, whereas the electron-donating substituents decrease the oxidation potential of these compounds. A broad oxidation peak was observed for **A5** which confirm a multi-steps of oxidation process. Whereas, SWV of **A3** and **A8** showed two anodic peaks due to two-steps oxidation, thus complementing CV results which appear as a broad oxidation peak. Moreover, I_pc_*vs* concentration plot is linear in lower concentration range (Example for **A7**: I_pa_ = 0.965 + 1.05 × 10^5^ X, R = 0.995) which reflects diffusion-controlled nature of the oxidation process, and at higher concentration plot shows a tendency to limit, which indicates the involvement of adsorption complication (Fig. 6).

## Conclusion

In conclusion, a series of indole-based-sulfonamide analogs (**A1**–**A8**) were synthesized and characterized by 1HNMR and HR-EI-MS. These compounds (**A1**–**A8**) can be obtained directly from the reactions of 5-fluoro-1H-indole-3-carbohydrazide (**I**) with different aryl sulfonyl chloride in the presence of pyridine. The electrochemical behavior of these new compounds **(A1**–**A8)** has been studied using CV and SWV at ambient temperature on a PGE. The obtained electrochemical results provide useful information such as redox behavior, electron affinity, oxidation potential, electrochemical parameters, mechanism and the stability of these electroactive derivatives. Structure electrochemical activity relationship has been also reviewed for all compounds, which shows that the nature, position and number of substituents on benzene ring play an important role.

## Experimental section

### General experimental

NMR experiments were performed on Ultra Sheild Bruker FT NMR 500 MHz. IR experiments were performed on Perkin Elmer FT-IR and UV, Perkin Elmer Lambda 35 UV–VIS Spectrometer. CHN analysis was performed on a Carlo Erba Strumentazione-Mod-1106, Italy. Ultraviolet Electron impact mass spectra (EI MS) were recorded on a Finnigan MAT-311A, Germany. Thin layer chromatography (TLC) was performed on pre-coated silica gel aluminum plates (Kieselgel 60, 254, E. Merck, Germany). Chromatograms were visualized by UV at 254 and 365 nm.

### Procedure for the synthesis 5-fluoro-1H-indole-3-carbohydrazide

The methyl 5-fluoro-1H-indole-3-carboxylate (10 g) was heated under reflux with hydrazine hydrated (10 mL) and methanol (25 mL) mixture for 6 h. The hydrazine and methanol were evaporated to get crude product that was recrystallized in ethanol and obtained pure 5-fluoro-1H-indole-3-carbohydrazide. Yield: 89%; ^1^HNMR (500 MHZ, DMSO-*d*_*6*_): *δ* 12.2 (s, 1H, NH), 11.24 (s, 1H, NH), 7.43 (d, *J* = 7.0 Hz, 1H, Ar), 7.35 (t, *J* = 7.0 Hz, 1H, Ar) 7.29 (dd, *J* = 7.0, 6.5 Hz, 1H, Ar), 7.22 (s, 1H, Ar), 4.26 (s, 2H, NH_2_); ^13^CNMR (125 MHZ, DMSO-*d*_*6*_): *δ* 163.9, 157.4, 131.2, 130.5, 127.1, 113.2, 112.3, 112.0, 111.9; HREI-MS: m/z calcd for C_9_H_8_FN_3_O, [M]+ 193.0651; Found; 193.0640.

### General procedure for the synthesis indole-based-sulfonamide derivatives

The indole-based-sulfonamide derivatives were synthesized by heating under reflux a mixture of 1 mmol each 5-fluoro-1H-indole-3-carbohydrazide and aryl-sulfonyl chloride in 10 mL Pyridine 2 h. The development of reaction checked by TLC. After accomplishment of reaction, the solvent was evaporated by vacuum to afford crude products which were further recrystallized in ethanol and got pure product in 85–78 yields.

#### *N*′-(5-Fluoro-*1H*-indole-3-carbonyl)benzenesulfonohydrazide (A1)

Yield: 82%; ^1^HNMR (500 MHZ, DMSO-*d*_*6*_): *δ* 12.19 (s, 1H, NH), 11.40 (s, 1H, NH), 11.10 (s, 1H, NH), 7.92–7.89 (m, 2H, Ar), 7.46–7.42 (m, 3H, Ar), 7.42 (d, *J* = 7.0 Hz, 1H, Ar), 7.31 (t, *J* = 7.0 Hz, 1H, Ar) 7.29 (dd, *J* = 7.5, 6.0 Hz, 1H, Ar), 7.26 (s, 1H, Ar); ^13^CNMR (125 MHZ, DMSO-*d*_*6*_): *δ* 164.5, 157.5, 139.4, 132.2, 131.0, 130.4, 129.0, 129.0, 127.5, 127.0, 127.0, 113.4, 112.5, 112.0, 111.8; HREI-MS: m/z calcd for C_15_H_12_FN_3_O_3_S, [M] + 333.0583; Found; 333.0565; Anal. Calcd for, C_15_H_12_FN_3_O_3_S; C, 54.05; H, 3.63; N, 12.61; Found: C, 54.01; H, 3.59; N, 12.54.

#### *N*′-(5-Fluoro-*1H*-indole-3-carbonyl)-4-nitrobenzenesulfonohydrazide (A2)

Yield:80%; ^1^HNMR (500 MHZ, DMSO-*d*_*6*_): *δ* 12.10 (s, 1H, NH), 11.20 (s, 1H, NH), 11.09 (s, 1H, NH), 8.42 (d, *J* = 8.0 Hz, 2H, Ar), 8.09 (d, *J* = 8.0 Hz, 2H, Ar), 7.41 (d, *J* = 7.0 Hz, 1H, Ar), 7.32 (t, *J* = 7.0 Hz, 1H, Ar) 7.27 (dd, *J* = 7.5, 6.0 Hz, 1H, Ar), 7.24 (s, 1H, Ar); ^13^CNMR (125 MHZ, DMSO-*d*_*6*_): *δ* 164.5, 157.6, 151.4, 145.5, 131.0, 130.5, 128.0, 128.0, 127.5, 127.5, 121.2, 113.5, 112.5, 112.1, 111.9; HREI-MS: m/z calcd for C_15_H_11_FN_4_O_5_S, [M]+ 378.0434; Found; 378.0422; Anal. Calcd for, C_15_H_11_FN_4_O_5_S; C, 43.91; H, 2.70; N, 13.65; Found: C, 43.84; H, 2.66; N, 13.61.

#### *N*′-(5-Fluoro-*1H*-indole-3-carbonyl)-3-nitrobenzenesulfonohydrazide (A3)

Yield:80%; ^1^HNMR (500 MHZ, DMSO-*d*_*6*_): *δ δ* 12.42 (s, 1H, NH), 11.26 (s, 1H, NH), 11.30 (s, 1H, NH), 8.60 (d, *J* = 2.0 Hz, 1H, Ar), 8.29 (d,d *J* = 8.0, 2.0 Hz, 1H, Ar), 8.19 (d,d *J* = 7.0, 2.0 Hz, 1H, Ar), 7.80 (t, *J* = 7.5 Hz, 1H, Ar), 7.43 (d, *J* = 7.0 Hz, 1H, Ar), 7.31 (t, *J* = 7.0 Hz, 1H, Ar) 7.26 (dd, *J* = 7.5, 6.0 Hz, 1H, Ar), 7.22 (s, 1H, Ar); ^13^CNMR (125 MHZ, DMSO-*d*_*6*_): *δ* 164.6, 157.3, 148.4, 140.3, 133.2, 131.1, 130.6, 130.1, 127.60, 124.1, 120.4, 113.6, 112.4, 112.0, 111.9; HREI-MS: m/z calcd for C_15_H_11_FN_4_O_5_S, [M] + 378.0434; Found; 378.0440; Anal. Calcd for, C_15_H_11_FN_4_O_5_S; C, 43.91; H, 2.70; N, 13.65; Found: C, 43.83; H, 2.64; N, 13.59.

#### 4-Fluoro-*N*′-(5-fluoro-*1H*-indole-3-carbonyl)benzenesulfonohydrazide (A4)

Yield:80%; ^1^HNMR (500 MHZ, DMSO-*d*_*6*_): *δ* 12.30 (s, 1H, NH), 11.55 (s, 1H, NH), 11.19 (s, 1H, NH), 7.89 (d, *J* = 8.5 Hz, 2H, Ar), 7.40 (d, *J* = 7.0 Hz, 1H, Ar), 7.31 (t, *J* = 7.0 Hz, 1H, Ar) 7.28 (dd, *J* = 7.5, 6.0 Hz, 1H, Ar), 7.25 (t, *J* = 8.5 Hz, 2H, Ar), 7.22 (s, 1H, Ar); ^13^CNMR (125 MHZ, DMSO-*d*_*6*_): *δ* 166.0, 164.5, 157.5, 135.2, 131.2, 130.5, 128.6, 128.6, 127.5, 115.7, 115.7, 113.7, 12.2, 112.0, 111.9; HREI-MS: m/z calcd for C_15_H_11_F_2_N_3_O_3_S, [M]+ 351.0489; Found; 351.0470; Anal. Calcd for, C_15_H_11_F_2_N_3_O_3_S; C, 51.28; H, 3.16; N, 11.96; Found: C, 51.22; H, 3.12; N, 11.91.

#### *N*′-(5-Fluoro-*1H*-indole-3-carbonyl)-4-methoxybenzenesulfonohydrazide (A5)

Yield: 84%; ^1^HNMR (500 MHZ, DMSO-*d*_*6*_): *δ* 12.60 (s, 1H, NH), 11.50 (s, 1H, NH), 11.30 (s, 1H, NH), 7.81 (d, *J* = 8.0 Hz, 2H, Ar), 7.41 (d, *J* = 7.0 Hz, 1H, Ar), 7.33 (t, *J* = 7.0 Hz, 1H, Ar) 7.27 (dd, *J* = 7.5, 6.0 Hz, 1H, Ar), 7.20 (s, 1H, Ar), 7.02 (d, *J* = 8.0 Hz, 2H, Ar), 3.82 (s, 3H, CH_3_); ^13^CNMR (125 MHZ, DMSO-*d*_*6*_): *δ* 164.7, 163.8, 157.4, 132.0, 131.2, 130.6, 128.0, 128.0, 127.5, 114.2, 114.2, 113.6, 112.4, 112.1, 111.9, 55.8; HREI-MS: m/z calcd for C_16_H_14_FN_3_O_4_S, [M] + 363.0689; Found; 363.0662; Anal. Calcd for, C_16_H_14_FN_3_O_4_S; C, 52.89; H, 3.88; N, 11.56; Found: C, C, 52.82; H, 3.83; N, 11.52.

#### 4-Cyano-*N’*-(5-fluoro-*1H*-indole-3-carbonyl)benzenesulfonohydrazide (A6)

Yield: 84%; ^1^HNMR (500 MHZ, DMSO-*d*_*6*_): *δ* 12.20 (s, 1H, NH), 11.70 (s, 1H, NH), 11.16 (s, 1H, NH), 8.10 (d, *J* = 7.5 Hz, 2H, Ar), 7.78 (d, *J* = 7.5 Hz, 2H, Ar), 7.42 (d, *J* = 7.5 Hz, 1H, Ar), 7.31 (t, *J* = 7.5 Hz, 1H, Ar) 7.28 (dd, *J* = 70, 6.0 Hz, 1H, Ar), 7.20 (s, 1H, Ar); ^13^CNMR (125 MHZ, DMSO-*d*_*6*_): *δ* 164.8, 157.4, 144.1, 132.3, 132.3, 131.0, 130.5, 128.1 128.1, 127.4, 115.6, 114.9, 114.9, 113.6, 112.6, 112.2, 111.8; HREI-MS: m/z calcd for C_16_H_11_FN_4_O_3_S, [M]+ 358.0536; Found; 358.0522; Anal. Calcd for, C_16_H_11_FN_4_O_3_S; C, 55.32; H, 4.06; N, 12.10; Found: C, 55.27; H, 4.01; N, 12.04.

#### *N’*-(5-Fluoro-*1H*-indole-3-carbonyl)-3,4-dimethoxybenzenesulfonohydrazide (A7)

Yield: 84%; ^1^HNMR (500 MHZ, DMSO-*d*_*6*_): *δ* 12.82 (s, 1H, NH), 11.90 (s, 1H, NH), 11.28 (s, 1H, NH), (d, *J* = 7.5 Hz, 2H, Ar), 7.42 (d, *J* = 7.5 Hz, 1H, Ar),7.36 (d, *J* = 7.0 Hz, 2H, Ar), 7.32 (d, *J* = 2.0 Hz, 2H, Ar), 7.29 (t, *J* = 7.5 Hz, 1H, Ar) 7.26 (dd, *J* = 70, 6.0 Hz, 1H, Ar), 7.21 (s, 1H, Ar), 6.91 (d, *J* = 8.0 Hz, 2H, Ar); ^13^CNMR (125 MHZ, DMSO-*d*_*6*_): *δ* 164.6, 157.4, 153.1, 150.0, 133.2, 131.5, 130.4, 127.5, 120.5, 115.5, 113.6, 1128, 112.5, 112.0, 111.9, 56.4, 56.2; HREI-MS: m/z calcd for C_17_H_16_FN_3_O_5_S, [M]+ 93.0795; Found; 393.0778; Anal. Calcd for, C_17_H_16_FN_3_O_5_S; C, C, 51.90; H, 4.10; N, 10.68; Found: C, 51.83; H, 4.06; N, 10.63.

#### *N’*-(5-Fluoro-*1H*-indole-3-carbonyl)-4-isopropylbenzenesulfonohydrazide (A8)

Yield: 84%; ^1^HNMR (500 MHZ, DMSO-*d*_*6*_): *δ* 12.92 (s, 1H, NH), 11.86 (s, 1H, NH), 11.53 (s, 1H, NH), 7.82 (d, *J* = 8.0 Hz, 2H, Ar), 7.41 (d, *J* = 7.5 Hz, 1H, Ar), 7.38 (d, *J* = 8.0 Hz, 2H, Ar), 7.33 (t, *J* = 7.5 Hz, 1H, Ar) 7.29 (dd, *J* = 70, 6.0 Hz, 1H, Ar), 7.22 (s, 1H, Ar), 2.72 (m, 1H, CH), 1.26 (d,, *J* = 10 Hz, 6H, 2XCH_3_); ^13^CNMR (125 MHZ, DMSO-*d*_*6*_): *δ* 164.7, 157.6, 151.2, 136.7, 131.3, 130.7, 127.6, 127.1 127.1, 126.5, 126.5, 113.6, 112.7, 112.4, 111.9, 36.2, 23.8, 23.8; HREI-MS: m/z calcd for C_18_H_18_FN_3_O_3_S, [M]+ 75.1053; Found; 375.1040; Anal. Calcd for, C_18_H_18_FN_3_O_3_S; C, C, 57.59; H, 4.83; N, 11.19; Found: C, 57.53; H, 4.79; N, 11.14.

### Voltammetric parameters and electrochemical cells

Voltammetric experiments, including cyclic voltammetry (CV) and square-wave voltammetry (SWV), were performed using an interface 1010E Potentiostat/Galvanostat/ZRA model (Gamry Instruments, Warminster, USA). The electrode system consisted of a pencil graphite electrode (PGE) as the working electrode, Ag/AgCl (saturated KCl) reference electrode, and a Pt wire auxiliary electrode. Operating conditions for SWV were frequency 50 Hz, pulse height 25 mVpp, scan increment 6 mV, and accumulation time 60 s. A Roca mechanical pencil Model M205R (Korea) was used as a holder for the pencil lead. Electrical contact with the lead was achieved by soldering a copper wire to the metallic part that holds the lead in place inside the pencil. The tested pencil leads were from Faber-Castell and named Super-polymer 9065S (black lead) of types 2B. All leads had a total length of 60 mm and a diameter of 0.5 mm. Immersing 10 mm of the pencil lead into a solution resulted in an active electrode area of 15.9 mm^2^. The pencil leads were used as received.

### Voltammetric reagents and materials

A stock solution of sulfonamide compounds was prepared by dissolving an appropriate amount of each compound in ethanol and storing the solution in the dark at 4 °C. Phosphate buffer solution (PBS) was prepared using the adequate amount of Na_2_HPO_4_ and NaH_2_PO_4_ salts. The pH of the aqueous solutions was measured using a pH-meter (Model pH-M-31114 Molequle-on) with accurate to ± 0.02 unit. All the chemicals were of reagent grade (Merck, Darmstadt). All solutions were prepared using water purified in a Millipore Milli-Q direct 8/16 system. All the experiments were carried out at room temperature (24 ± 2 °C) and, to eliminate oxygen, N_2_ was bubbled into the solution for 5 min.

## Supplementary information

**Additional file 1: Fig. S1.** CVs (scan 1–10) of 3.0 x 10^−5^ M of **A5** (a) and **A3** (b) in PBS of pH 7.4 obtained at PGE scan rate, 0.10 Vs^−1^. **Fig. S2.** CVs of 3.0 x 10^−5^ M of **A1**–**A8** in PBS of pH 3.0 obtained at PGE scan rate, 0.10 Vs^−1^. **Fig. S3.** Plot of log I_pa_ vs. log ν of **A1**–**A8** in PBS of pH 7.4 obtained from CV at PGE. **Fig. S4.** Plot of E_p_ (V) vs ln ν (mV/s) of **A1**–**A8** in PBS of pH 7.40 obtained from CV at PGE.

## Data Availability

The datasets used and/or analysed during the current study available from the corresponding author on reasonable request.

## Abbreviations

CV: Cyclic voltammetry
SWV: Square wave voltammetry
PGE: Pencil graphite electrode
k_s_: Standard heterogeneous rate constants
Г: Electroactive surface coverage
PBS: Phosphate buffer solution
ν: Scan rate
α: Electron transfer coefficient
E_o_: Formal redox potential
*Q*: Charge involved in the electrooxidation process
n: Number of electrons
F: Faraday constant
A: Working electrode surface area
EI MS: Electron impact mass spectra
TLC: Thin layer chromatography
