# Supplementary material for: Synthesis, characterization and electrochemical properties of some biologically important indole-based-sulfonamide derivatives
Source: BMC Chem. 2020 May 27;14(1):38. doi: 10.1186/s13065-020-00691-5 (PMC7254745; doi:10.1186/s13065-020-00691-5)
Supplement: Supplementary file 1 — Additional file 1: Fig. S1. CVs (scan 1–10) of 3.0 x 10^−5^ M of A5 (a) and A3 (b) in PBS of pH 7.4 obtained at PGE scan rate, 0.10 Vs−1. Fig. S2. CVs of 3.0 x 10^−5^ M of A1–A8 in PBS of pH 3.0 obtained at PGE scan rate, 0.10 Vs−1. Fig. S3. Plot of log Ipa vs. log ν of A1–A8 in PBS of pH 7.4 obtained from CV at PGE. Fig. S4. Plot of Ep (V) vs ln ν (mV/s) of A1–A8 in PBS of pH 7.40 obtained from CV at PGE. [file 13065_2020_691_MOESM1_ESM.docx]

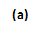

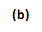

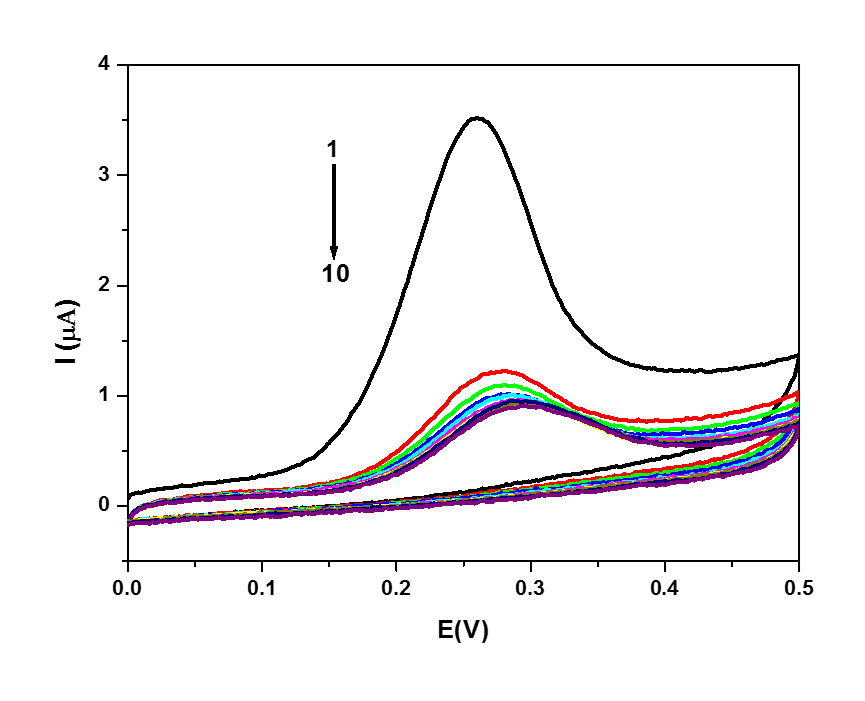


**Fig. S1.** CVs (scan 1–10) of 3.0x10^-5^ M of **A5** (a) and **A3** (b) in PBS of pH 7.4 obtained at PGE scan rate, 0.10 Vs^-1^.

**Fig. S2.** CVs of 3.0x10^-5^ M of **A1-A8** in PBS of pH 3.0 obtained at PGE scan rate, 0.10 Vs^-1^.

** Fig. S3.** Plot of log I_pa_ vs. log ν of **A1-A8** in PBS of pH 7.4 obtained from CV at PGE.

**Ln ν (mV/s)**

**Fig. S4.** Plot of E_p_ (V) vs ln ν (mV/s) of **A1-A8** in PBS of pH 7.40 obtained from CV at PGE.
